# Enhancing the Stability of ^211^At Radiopharmaceuticals: Insights from Ortho-Substituent Strategies

**DOI:** 10.1021/acsmedchemlett.5c00102

**Published:** 2025-03-20

**Authors:** Taoqian Zhao, Steven H. Liang

**Affiliations:** Department of Radiology and Imaging Sciences, 1371 Emory University, Atlanta, Georgia 30322, United States

**Keywords:** Targeted alpha therapy (TAT), Astatine-211 (^211^At), Radiopharmaceuticals, Radiochemistry

## Abstract

Astatine-211 (^211^At) is a promising alpha-emitting radionuclide for targeted alpha therapy (TAT), delivering high linear energy transfer (LET) and a short radiation range, making it ideal for cancer treatment while minimizing damage to surrounding healthy tissue. This viewpoint highlights recent advancements in the development of astatine-211 compounds for TAT, with a focus on the role of neighboring substituents in enhancing in vivo stability. By mitigating deastatination, these structural modifications improve radiopharmaceutical integrity, paving the way for more effective and clinically viable ^211^At-based radiopharmaceuticals.

## Graphical abstract

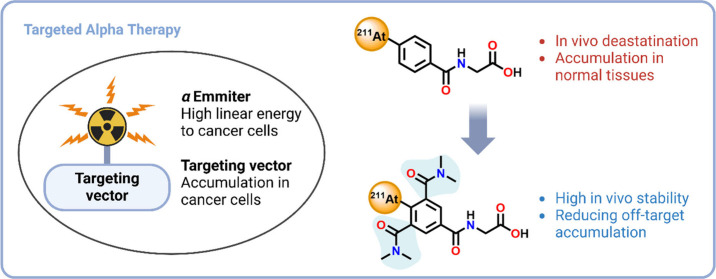

Targeted alpha therapy (TAT) has emerged as a promising strategy for the precise treatment of cancer, leveraging the high cytotoxicity and short-range tissue penetration of alpha particles to minimize off-target effects.^1,2^ TAT radiopharmaceuticals are composed of two key components: an alpha-emitting radionuclide and a targeting vector.^3^ The alpha-emitting radionuclide delivers high linear energy transfer (LET) radiation, inducing efficient DNA double-strand breaks and subsequent cell death.^4^ The targeting vector ensures selective tumor accumulation by binding to tumor-associated proteins while minimizing off-target effects on healthy tissues. A wide range of targeting vectors has been investigated, including peptides,^5^ antibodies,^6^ and small molecules,^7^ each offering distinct advantages in terms of binding specificity, pharmacokinetics, and tumor penetration.

Astatine-211 (^211^At) is a particularly attractive radionuclide for TAT attributed to its favorable physical and radiobiological properties.^8^ With a half-life of approximately 7.2 h, ^211^At decays primarily via alpha emission (42%) and electron capture (58%), enabling highly effective localized tumor destruction while limiting long-range radiation damage. The production of ^211^At is achieved through cyclotron-based nuclear reactions, where a bismuth-209 (^209^Bi) target is bombarded with alpha particles), leading to the reaction:

B209i+α→211At+2n

Following its production, ^211^At undergoes a decay cascade that influences its radiopharmaceutical applications. It primarily decays to bismuth-207 (^207^Bi) through electron capture, while a fraction undergoes alpha decay to polonium-211 (^211^Po), which subsequently emits another α particle to form lead-207 (^207^Pb), a stable end product (Figure 1).^9^ This decay pathway ensures efficient tumor irradiation with minimal long-lived radioactive residues, making ^211^At a compelling choice for clinical alpha-particle radiotherapy.^10^

**Figure 1:**
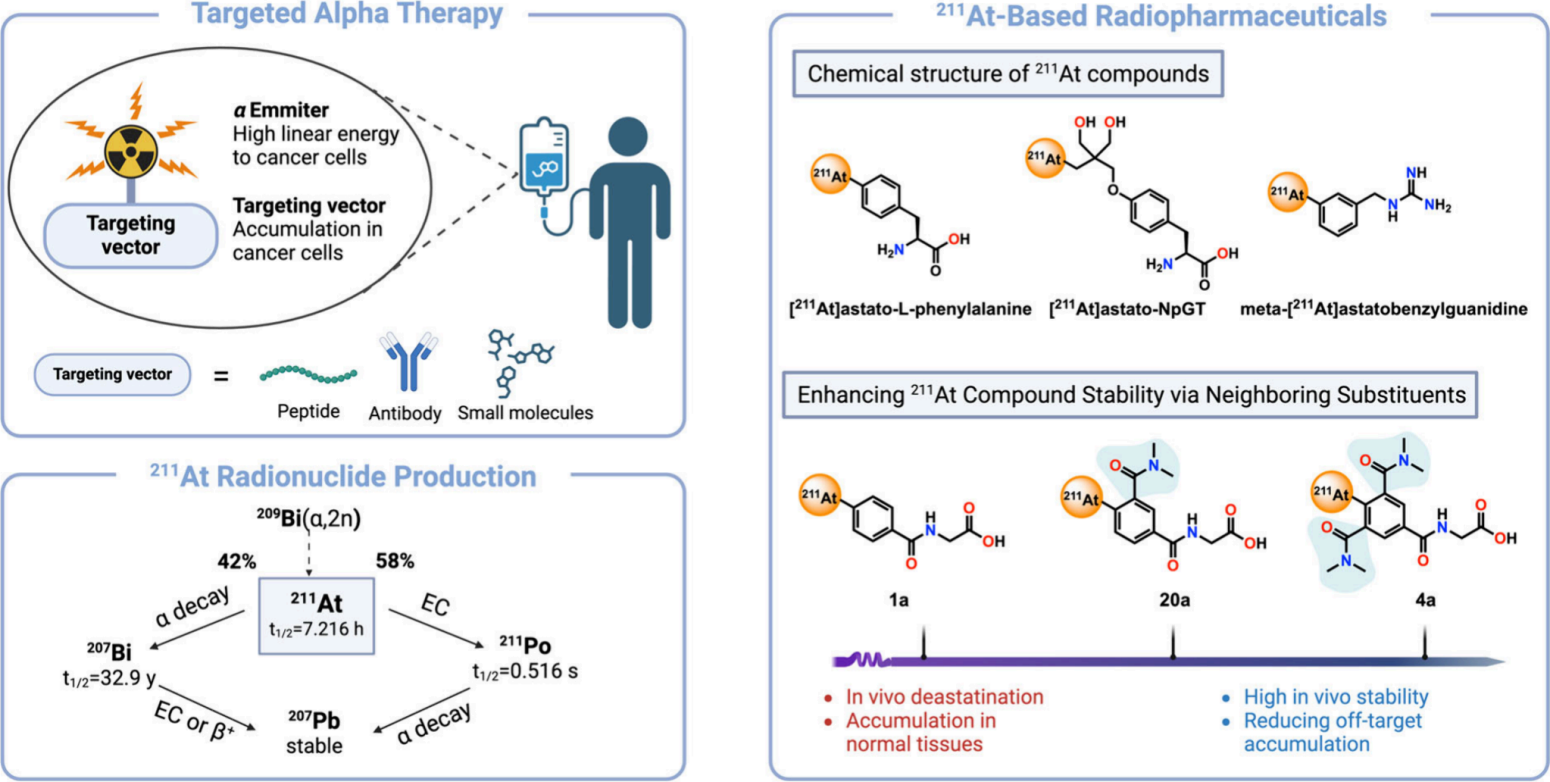
Overview of targeted alpha therapy and ^211^At-based radiopharmaceuticals highlighting their mechanisms, decay pathways, and therapeutic applications. Abbreviations: **EC**, electron capture; **β** ^**+**^, positron emission; **t**_**1**_ **/**_**2**_, half-life.

The development of ^211^At-labeled radiopharmaceuticals has advanced considerably, with ongoing efforts focused on improving stability, tumor specificity, and therapeutic efficacy. Given its halogen-like properties, ^211^At can be incorporated into both small molecules and biomolecules (e.g., antibodies and peptides), offering versatility in TAT.^9^ However, its clinical translation has been hindered by the in vivo instability of the carbon–astatine bond, leading to deastatination and nonspecific accumulation.^11^ To address this challenge and harness the full potential of ^211^At, several ^211^At-labeled compounds have been developed with innovative chemical modifications to improve in vivo stability (Figure 1). [^211^At]­astato-*L*-phenylalanine, an amino acid derivative, benefits from an optimized molecular structure that enhances tumor uptake while reducing deastatination.^12^ Astato-*N*-[2-(3-NNN-guanidino)­propyl]­tyrosine ([^211^At]­astato-NpGT) incorporates two hydroxyl groups, which act as stabilizers, significantly improving its resistance to in vivo degradation.^13^ Meanwhile, *meta*-[^211^At]­astatobenzylguanidine (m-[^211^At]­ABG), an analog of *meta*-iodobenzylguanidine (MIBG), demonstrates enhanced stability and selective tumor targeting, making it a strong candidate for treating neuroendocrine and adrenergic tumors such as neuroblastoma and pheochromocytoma.^14^ These advancements highlight the critical role of structural modifications in overcoming the challenges of ^211^At radiopharmaceuticals, ensuring both effective tumor targeting and prolonged in vivo retention.

These ^211^At-based radiopharmaceuticals exemplify the potential of alpha-emitting isotopes in precision oncology. The combination of its favorable decay characteristics, targeted delivery, and therapeutic potential underscores its growing role in advancing radiopharmaceutical therapy.

Based on previous research aimed at improving the stability of ^211^At-labeled radiopharmaceuticals, a recent study systematically evaluated the biological stability of astatinated hippuric acid derivatives, with a particular emphasis on the role of neighboring substituents in modulating radiopharmaceutical stability and in vivo behavior.^15^

To assess stability, in vitro assays were conducted, as summarized in Table 1. The findings demonstrated that astatinated compounds lacking adjacent substituents underwent minor deastatination, resulting in reduced radiochemical purity over time. In contrast, compounds incorporating two ortho-dimethylcarbamoyl substituents maintained over 90% purity after 1 h of incubation at 37 °C, highlighting their superior stability.

**Table 1:** Stability of Compounds in Murine Plasma Following Incubation at 37°C for 1 h

	**1**	**2**	**3**	**4**
^211^At	81.4 (1.60)	88.7 (0.64)	81.5 (2.00)	92.0 (1.85)
^125^I	98.4 (0.31)	95.8 (1.97)	92.9 (0.46)	92.7 (1.68)

Analysis of plasma samples using HPLC and TLC. Data represents the remaining percentage (%) of each compound, expressed as the mean (SD) from three independent samples. For detailed structural representations, refer to Figure 2.

**Figure 2:**
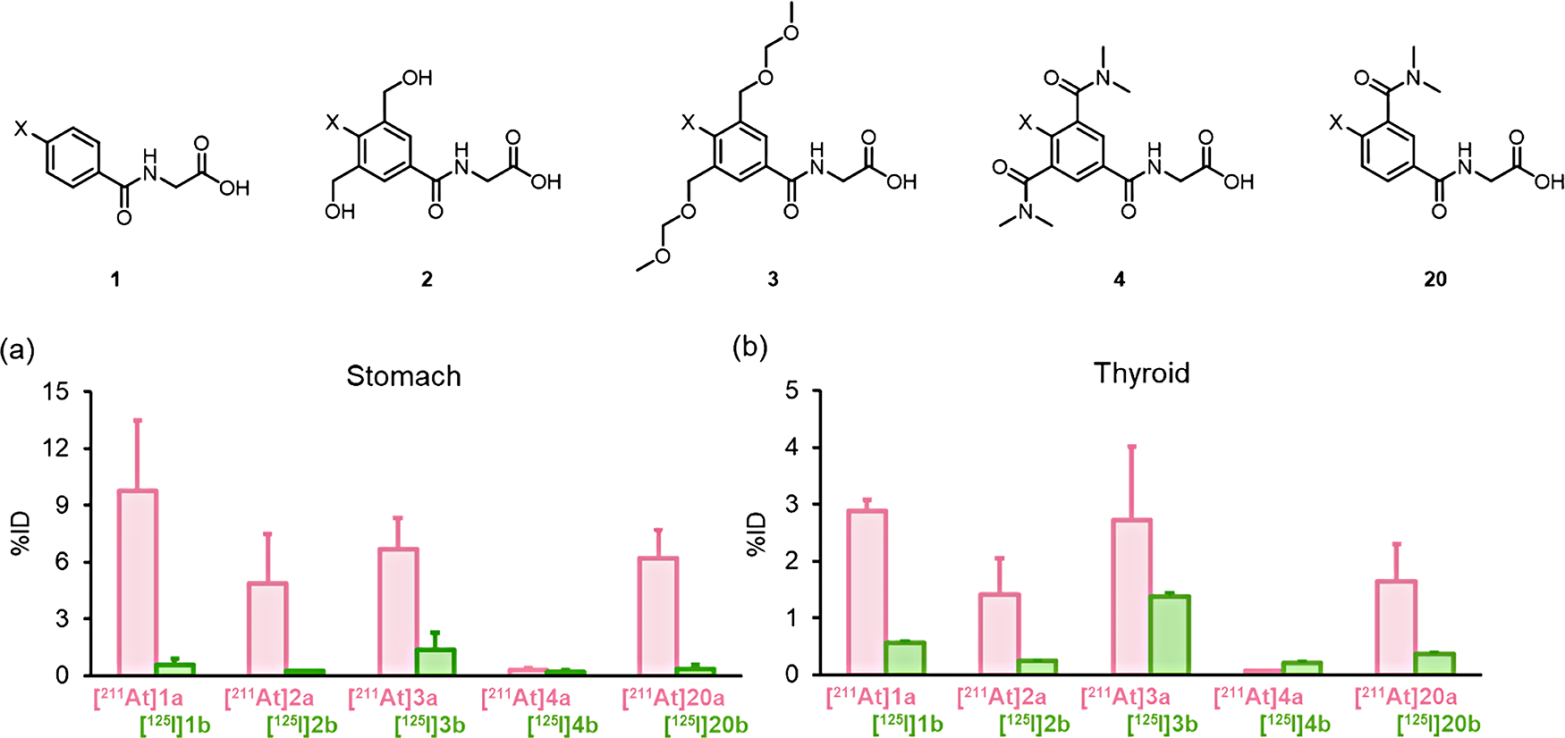
Summary of radioactivity accumulation in (a) the stomach and (b) the thyroid at 4 h postintravenous injection of ^211^At-labeled and ^125^I-labeled compounds in healthy mice. Reproduced with permission from ref 15. Copyright 2024 American Chemical Society.

Figure 2 provides a comparative assessment of in vivo radioactivity accumulation in the stomach and thyroid of mice across all evaluated compounds, offering valuable insights into their relative stability and biodistribution. These findings revealed that a single ortho-dimethylcarbamoyl group provided stability comparable to nonortho-substituted compounds but was significantly less effective than two ortho-dimethylcarbamoyl groups. This study highlights the critical role of neighboring substituents in stabilizing ^211^At-labeled compounds and provides valuable insights for optimizing radiopharmaceutical design to enhance clinical viability.

The development of ^211^At radiopharmaceuticals continues to advance, offering great promise for enhancing TAT, especially in the treatment of micrometastatic and therapy-resistant tumors. While substantial progress has been made in stabilizing astatinated compounds through strategic substituent modifications, further refinements are needed to fully harness ^211^At’s therapeutic potential. Future research should prioritize optimizing radiochemical stability, refining linker chemistry, and broadening the application of biocompatible targeting vectors to enhance tumor selectivity and retention while minimizing off-target accumulation.

Looking forward, integrating ^211^At-based therapies with multimodal imaging techniques, such as PET imaging,^16–18^ along with personalized treatment strategies, could greatly enhance precision oncology by enabling real-time monitoring of therapeutic responses. Overcoming current challenges—particularly in radiochemical stabilization, molecular targeting, and imaging-guided therapy—will be essential for unlocking the full clinical potential of ^211^At. With continued innovation, ^211^At has the potential to expand its applications and improve the TAT efficacy.

## Abbreviations

TAT: targeted alpha therapy
LET: linear energy transfer
EC: electron capture
^211^At: astatine-211
m-[^211^At]­ABG: *meta*-[^211^At]­astatobenzylguanidine
MIBG: *meta*-iodobenzylguanidine
PET: positron emission tomography

## References

[1] Kim Y. S., Brechbiel M. W. (2012). An Overview of Targeted Alpha Therapy. Tumour Biol..

[2] Feuerecker B., Kratochwil C., Ahmadzadehfar H., Morgenstern A., Eiber M., Herrmann K., Pomykala K. L. (2023). Clinical
Translation
of Targeted α-Therapy: An Evolution or a Revolution?. J. Nucl. Med..

[3] Nelson B. J. B., Wilson J., Andersson J. D., Wuest F. (2023). Theranostic Imaging
Surrogates for Targeted Alpha Therapy: Progress in Production, Purification,
and Applications. Pharmaceuticals (Basel).

[4] Miederer M., Benešová-Schäfer M., Mamat C., Kästner D., Pretze M., Michler E., Brogsitter C., Kotzerke J., Kopka K., Scheinberg D. A., McDevitt M. R. (2024). Alpha-Emitting Radionuclides: Current Status and Future
Perspectives. Pharmaceuticals (Basel).

[5] Liu W., Ma H., Liang R., Chen X., Li H., Lan T., Yang J., Liao J., Qin Z., Yang Y., Liu N., Li F. (2022). Targeted Alpha Therapy of Glioma Using 211At-Labeled
Heterodimeric Peptide Targeting Both VEGFR and Integrins. Mol. Pharmaceutics.

[6] Pasternack J. B., Domogauer J. D., Khullar A., Akudugu J. M., Howell R. W. (2014). The Advantage
of Antibody Cocktails for Targeted Alpha Therapy Depends on Specific
Activity. J. Nucl. Med..

[7] Pallares R. M., Abergel R. J. (2022). Development of Radiopharmaceuticals
for Targeted Alpha
Therapy: Where Do We Stand?. Front. Med. (Lausanne).

[8] Feng Y., Zalutsky M. R. (2021). Production, Purification
and Availability of 211At:
Near Term Steps Towards Global Access. Nucl.
Med. Biol..

[9] Zalutsky M. R., Pruszynski M. (2011). Astatine-211:
Production and Availability. Curr. Radiopharm..

[10] Albertsson P., Bäck T., Bergmark K., Hallqvist A., Johansson M., Aneheim E., Lindegren S., Timperanza C., Smerud K., Palm S. (2023). Astatine-211 Based
Radionuclide Therapy: Current Clinical Trial Landscape. Front. Med. (Lausanne).

[11] Ayed T., Pilmé J., Tézé D., Bassal F., Barbet J., Chérel M., Champion J., Maurice R., Montavon G., Galland N. (2016). (211)­At-Labeled
Agents for Alpha-Immunotherapy: On
the In Vivo Stability of Astatine-Agent Bonds. Eur. J. Med. Chem..

[12] Watabe T., Kaneda-Nakashima K., Shirakami Y., Liu Y., Ooe K., Teramoto T., Toyoshima A., Shimosegawa E., Nakano T., Kanai Y., Shinohara A., Hatazawa J. (2020). Targeted Alpha Therapy Using Astatine (211At)-Labeled
Phenylalanine: A Preclinical Study in Glioma Bearing Mice. Oncotarget.

[13] Suzuki H., Kaizuka Y., Tatsuta M., Tanaka H., Washiya N., Shirakami Y., Ooe K., Toyoshima A., Watabe T., Teramoto T., Sasaki I., Watanabe S., Ishioka N. S., Hatazawa J., Uehara T., Arano Y. (2021). Neopentyl
Glycol as a Scaffold to Provide Radiohalogenated Theranostic Pairs
of High In Vivo Stability. J. Med. Chem..

[14] Sudo H., Tsuji A. B., Sugyo A., Nagatsu K., Minegishi K., Ishioka N. S., Ito H., Yoshinaga K., Higashi T. (2019). Preclinical Evaluation of the Acute Radiotoxicity of
the α-Emitting Molecular-Targeted Therapeutic Agent 211At-MABG
for the Treatment of Malignant Pheochromocytoma in Normal Mice. Transl. Oncol..

[15] Hirata S., Mishiro K., Washiyama K., Munekane M., Fuchigami T., Arano Y., Takahashi K., Kinuya S., Ogawa K. (2025). In Vivo Stability
Improvement of Astatobenzene Derivatives by Introducing Neighboring
Substituents. J. Med. Chem..

[16] Rong J., Haider A., Jeppesen T. E., Josephson L., Liang S. H. (2023). Radiochemistry for Positron Emission
Tomography. Nat. Commun..

[17] Deng X., Rong J., Wang L., Vasdev N., Zhang L., Josephson L., Liang S. H. (2019). Chemistry for Positron Emission Tomography:
Recent Advances in 11C-, 18F-, 13N-, and 15O-Labeling Reactions. Angew. Chem., Int. Ed. Engl..

[18] Krishnan H. S., Ma L., Vasdev N., Liang S. H. (2017). 18F-Labeling of Sensitive Biomolecules
for Positron Emission Tomography. Chemistry.

